# The limits of automatic sensorimotor processing during word processing: investigations with repeated linguistic experience, memory consolidation during sleep, and rich linguistic learning contexts

**DOI:** 10.1007/s00426-021-01620-4

**Published:** 2021-12-01

**Authors:** Fritz Günther, Sophia Antonia Press, Carolin Dudschig, Barbara Kaup

**Affiliations:** University of Tübingen, Tübingen, Germany

## Abstract

While a number of studies have repeatedly demonstrated an automatic activation of sensorimotor experience during language processing in the form of action-congruency effects, as predicted by theories of grounded cognition, more recent research has not found these effects for words that were just learned from linguistic input alone, without sensorimotor experience with their referents. In the present study, we investigate whether this absence of effects can be attributed to a lack of repeated experience and consolidation of the associations between words and sensorimotor experience in memory. To address these issues, we conducted four experiments in which (1 and 2) participants engaged in two separate learning phases in which they learned novel words from language alone, with an intervening period of memory-consolidating sleep, and (3 and 4) we employed familiar words whose referents speakers have no direct experience with (such as *plankton*). However, we again did not observe action-congruency effects in subsequent test phases in any of the experiments. This indicates that direct sensorimotor experience with word referents is a necessary requirement for automatic sensorimotor activation during word processing.

Language is one of our most important cognitive tools, and allows us to convey information across persons, space, and time. However, it is far from obvious how we can obtain meaning from the arbitrary visual and auditory patterns that form the symbols we call words. Proponents of the embodied cognition approach (e.g. Barsalou, 1999; Glenberg & Kaschak, 2002) have pointed out that language cannot be a self-contained system where the meaning of every symbol is defined by its relations to other symbols (an underlying assumption of other accounts; see Collins & Loftus, 1975; Fodor, 2000), as this would imply an infinite regress. This *symbol grounding problem* was illustrated by Harnad (1990) in a thought experiment where he argues that a monolingual English speaker will never be able to understand Chinese symbols using only a monolingual Chinese dictionary, with no reference to anything she understands (in this case, her native language). Analogously, the same argument can be made for our cognitive system: theories of embodied cognition argue that our cognitive system is only able to understand and assign meaning to the arbitrary symbols that are words if these are grounded in its primary functions—perception and action (Glenberg, 2015; Glenberg & Robertson, 2000; Harnad, 1990).

According to the experiential trace model (Zwaan and Madden, 2005), this grounding of word meanings is established through systematic and repeated co-occurrence between sensorimotor and linguistic experience (for example, hearing the word *balloon* while seeing a balloon in the sky), which establishes associative relations between the two (see also Hauk et al., 2004). The word *balloon* then serves as a cue to re-activate this sensorimotor experience, enabling the recipient to comprehend its meaning.

In line with this account, Lachmair et al. (2011) showed that vertical hand movement reactions to words describing a referent with a typical vertical location (*cloud* versus *basement*) were faster when the movement direction matched this implied location. Importantly, this was the case even when in a stroop-like task that did not require access to the word meaning (e.g., reacting towards the words’ font color), indicating automatic sensorimotor activation during word processing. This congruency effects for vertical hand movements during the processing of vertically related words have been well-replicated and firmly established in a series of studies (Dudschig et al., 2014a; Dudschig & Kaup, 2017; Thornton et al., 2013). Even more direct evidence for the predictions of the experiential trace model (Zwaan and Madden, 2005) was provided by Öttl et al. (2017), who observed this automatic congruency effect even for novel words, after participants learned them as labels for novel object referents presented in a specific vertical location.

However, we clearly know many words whose referents we never experienced directly. Even minimal exposure in language, such as reading the novel word *faller* in the sentences “The knight rode his loyal faller into battle.” and “The faller’s scales heat up as it is basking in the sun.” allows the reader to understand its meaning (Lazaridou et al., 2017)—in this case, most likely a large and fast reptile that is strong enough to carry a man in full armour. School children are estimated to learn the meaning of more than ten new words per day, without much change in their direct experience (Landauer & Dumais, 1997), and even adults learn new words on an almost daily basis (Brysbaert et al., 2016). How are we able to understand the meaning of these words, even if we just hear or read about them and the sensorimotor experience that is allegedly required to understand these meanings is missing?

A possible solution to this problem is that the meaning of such words could be grounded indirectly, via familiar words that are already grounded (for example, *faller* via the contextually-similar words *horse* and *lizard*, for which experience is available) (Harnad, 1990; Hoffman et al., 2018). In a series of four experiments, Günther et al. (2018) tested this hypothesis by having their participants learn novel words solely from language input which clearly implied a specific vertical location: They were learned in pairs with real words implying a vertical location (Ouyang et al., 2017), as replacements for these real words in natural sentences (Lazaridou et al., 2017), as new labels for these real words (compare Dudschig et al., 2014b), or as labels for novel concepts constructed from these real words (see Harnad, 1990). However, in subsequent test phases, no automatic action-congruency effect was observed for these novel words (contrary to Lachmair et al., 2011; Öttl et al., 2017), even though participants were perfectly able to explicitly indicate the words’ implied locations after the experiments.

How can this discrepancy between the results by Lachmair et al. (2011) (who observed automatic congruency effects for familiar words) on the one hand and Günther et al. (2018) (who observed no automatic congruency effects for newly learned words) on the other hand be explained? Since both studies employed the same behavioural paradigm except for the word material, the difference needs to arise from differences in the word material. The most straightforward explanation would be that novel words learned from language alone are simply not connected to sensorimotor experience and therefore cannot lead to an activation of said experience. However, a recent study by Günther et al. (2020) presents evidence against this explanation: Using the same learning phase as Günther et al. (2018) but a different task in the test phase—plausibility judgments for sentences including the novel words—these authors demonstrate action-congruency effects when the meaning of these novel words is accessed. This demonstrates that in principle the processing of these novel words can lead to the relevant sensorimotor activation, leaving open the question why this does not happen *automatically*, as it apparently does for familiar words related to a vertical location (Lachmair et al., 2011).

At this point, one can argue that a major difference still lies in the amount and quality of learning experience participants had with the novel compounds, which in the previous studies by Günther et al. (2018) was rather limited. We know that connections between experiential traces are assumed to be formed through *repeated* experience (Hauk et al., 2004; Zwaan and Madden, 2005), as is the case for associative learning in general. Furthermore, since learning was always immediately followed by the test phase, there was no possibility to consolidate the learned associations in memory. Indeed, it has been shown that night’s sleep plays an important role for memory consolidation (Walker & Stickgold, 2006), also specifically for the learning of new vocabulary (Henderson et al., 2012; Kurdziel et al., 2016). It stands to reason that, when the novel word meanings are properly learned and consolidated in memory—as has been the case for familiar words—the typical automatic congruency effects (Lachmair et al., 2011) can be expected.

We investigate this possibility in the present study. To this end, we adapted the general experimental paradigm by Günther et al. (2018), but implemented two critical changes which should both result in more natural learning scenarios: (a) the novel word learning phase was taking place in a semantically enriched context using vivid texts (see the Methods section for details), and (b) we introduced a memory consolidation phase during night’s sleep. If the previous studies only failed to observe action congruency effects due to limited associative strength between the words and sensorimotor experience, we expect to observe such effects in these experiments.

## Experiment 1

In the first experiment, the newly learned associative connections for the novel words can be consolidated in a phase of night’s sleep (Henderson et al., 2012; Kurdziel et al., 2016; Walker & Stickgold, 2006) between learning and the test phase. In addition, we also extended previous experiments (Günther et al., 2018) by employing an engaging learning task in which the use of novel words is warranted by the communicative context, as they serve as labels for new concepts in natural text.

### Method

#### Participants

The sample size for the current experiment was determined following the power analysis in Günther et al. (2018), based on the effect sizes of the action-congruency effects observed by Lachmair et al. (2011) and Öttl et al. (2017). The test power was estimated as power $\ge 0.90$ for sample sizes of $n \ge 42$ and $n \ge 38$, respectively. Since test power monotonically increases with sample size, Günther et al. (2018) decided to test 45 participants for all their experiments. We adopted this decision, setting our planned sample size to $n = 45$ for all experiments.

In Experiment 1, data were collected from 46 native German speaking participants (one more than required due to procedural issues), 36 female and 10 male, 39 right-handed, $M_{{\text {Age}}} = 22.3$ years, $SD_{{\text {Age}}} = 2.47$ years. We originally tested 52 participants, but data from two additional participants was excluded due to technical problems, and data from four additional participants was excluded due to high error rates (< 90% correct in at least one experimental condition; Lachmair et al., 2011). Participants in all experiments reported here received either money (at a rate of ${ } 8$ per hour for the active parts of the study) or course credit for their participation. No individual participated in more than one of the experiments or rating studies reported in this article.

#### Materials and procedures

Participants conducted the initial learning phase and sleep phase in the evening at home, followed by the repetition phase, test phase and explicit judgment task in a single lab session the next morning.

*Learning phase* Participants learned eight German-sounding pseudowords already employed in Günther et al. (2018) as well as Günther et al. (2020). Each word was embedded in one of eight texts (between 376 and 520 words), occurring between five and nine times. Considering the length of the texts, a number of eight learning items keeps the difficulty of the learning phase at a manageable level. The texts described a (slightly dystopian, futuristic) setting, before introducing one of the novel words referring to a novel concept within this setting. Four of the texts introduced upwards-related concepts (such as an artificial sun), and four introduced downwards-related concepts (such as an underground city). Examples for these texts are provided in Supplementary Material A.

This learning material was validated in a web-based rating study with 50 native German-speaking participants (37 female, 12 male, 1 not specified; $M_{{\text {Age}}} = 27.74$ years, $SD_{{\text {Age}}} = 8.40$ years). Novel words introduced as labels for upwards-related novel concepts which were correctly judged as upwards-related by between 70 and 88% of participants, and as downwards-related when describing downwards-related concepts by between 80 and 86% of participants (all significantly different from 50%, $p < 0.007$). In the actual experiment, four of the novel words were used in texts describing upwards-related novel concepts, and the other four for the downwards-related novel concepts. For 22 of the 46 participants, this assignment between novel words and text was reversed. In the first, web-based learning phase (between 8 pm and 10 pm), participants were instructed to carefully read the texts 12 h before their lab session started. The texts were presented in random order, and participants could proceed to the next text at their choosing. All web-based parts of the study were programmed using *jsPsych* (de Leeuw, 2015). The starting time and end time of the learning phase, as well as the presentation time of each text were logged. At the end of the learning phase, participants generated an individualized code to confirm their participation.

*Sleep phase* Participants were instructed to sleep between the learning phase and their lab session the next morning, and to engage in as little activities as possible apart form sleeping, especially no other learning activities. Participants reported sleep durations between 4 h 15 min and 9 h 30 min.

*Repetition phase* In the lab session starting between 8 am and 10 am, it was initially checked if and when participants performed the learning phase, by asking them to provide their individualized code and by inspecting whether they completed the learning phase within a reasonable time frame. Participants then read their learning phase texts a second time.

*Test phase* The test phase was identical to Günther et al. (2018) and Öttl et al. (2017). Participants were seated in front of a computer monitor and a vertically mounted computer keyboard with a special four-button overlay (two buttons in the middle, one above the other, one upper button, and one lower button).

Participants started each trial by pressing the two middle buttons of the keyboard. Half of the participants were instructed to press the upper middle button with their dominant hand, half the lower middle button. When both buttons were pressed at the same time, a blank screen appeared for 1000 ms, followed by a black fixation cross in the center of the screen for 750 ms. Then, one of the eight novel words was presented in the center of the screen in one of four font colors (blue, red, orange or green). Participants were instructed to react with an upwards movement (release the upper middle button and press the upper button with the same hand) for two of the colors and a downwards movement for the other two (Lachmair et al., 2011, see also Dudschig et al., 2014a, b). The assignment of response directions to colors was counterbalanced across participants. Response time is measured as the time until one of the middle buttons is released (Lachmair et al., 2011).^1^ The word disappeared when one of the middle buttons was released, or after a fixed duration of 1500 ms. Participants received feedback if their answer was incorrect or too slow.

Each of the eight experimental blocks consisted of 32 trials (8 novel words, all presented in each of the 4 colors). Before the actual test phase, participants completed a practice block of 16 trials, in which two different letter strings (XXXX and YYYY) were presented to the participants twice in each font color. The test phase was implemented in *Psychtoolbox* for Matlab (Brainard, 1997).

*Explicit judgment task* In the explicit judgment task directly following the test phase, participants indicated for each novel words whether they associated it with an upwards or a downwards location (as in Günther et al., 2018).

### Results

All data and analysis scripts (as well as the experimental material) for this and all experiments are available at https://osf.io/vxrhn.

#### Test phase

**Fig. 1:**
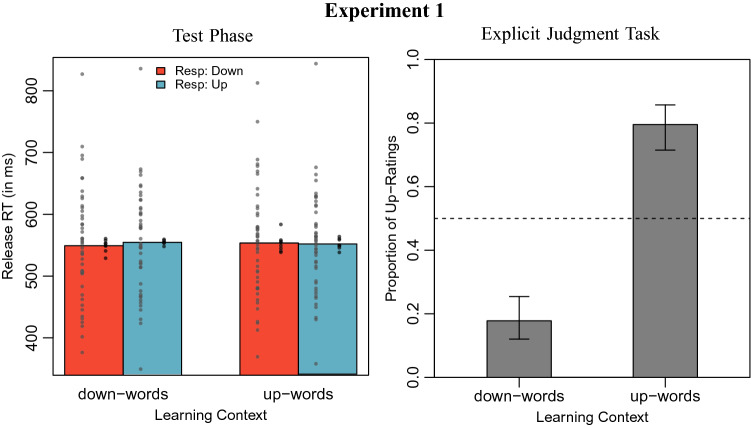
*Left panel* Mean release times in Experiment 1, by learning context and response direction. The grey circles on the left of each bar display mean reaction times by participants, the black circles on the right of each bar display mean reaction times by item. *Right panel* Proportion of upwards-location ratings for novel words by learning context, with 0.95 confidence intervals

Error trials (2.9 %) and overly fast trials (RT < 100 ms, 2 trials) were excluded from analysis (Lachmair et al., 2011). Mean response times by learning context and response direction are displayed in Fig. 1.

We employed linear mixed effect models to analyze log-transformed reaction times (Baayen and Milin, 2010), using the R packages *lme4* (Bates et al., 2015) and *lmerTest* (Kuznetsova et al., 2017). We first estimated a baseline model including fixed effects for learning context and response direction, random intercepts for both participants and items, and random slopes for learning context and response direction for both participants and items.^2^ Additionally including a fixed effect interaction between learning context and response direction (corresponding to the hypothesized action-congruency effect) did not improve the model, as indicated in a model comparison via likelihood-ratio test ($\chi ^2(1) = 1.78, p = 0.183$). Using the BIC approximation BF $= \exp ({\text {BIC}}(H_1) - {\text {BIC}}(H_0)/2)$ (Wagenmakers, 2007), we obtained a Bayes factor of BF $= 0.0227$ for this comparison, indicating that the data are about 44 times more likely under the baseline model (strong evidence in favor of the baseline/null model; Kass & Raftery, 1995).

The same pattern emerged when restricting the analysis to items for which participants gave the correct answer in the explicit judgment task. The model parameters for the model including the interaction term are reported in Table 1.

**Table 1:** Model parameters for the model including a fixed effect for the interaction between learning context and response direction in Experiment 1 and Experiment 2

Experiment	Parameter	$\beta$	*t*	*p*
Experiment 1	Intercept	6.27	264.65	< 0.001
	Learning context: up	0.01	1.28	0.203
	Response direction: up	0.01	1.07	0.291
	Learn. con.: up $\times$ resp. dir.: up	− 0.01	− 1.33	0.183
Experiment 2	Intercept	6.21	322.04	< 0.001
	Learning context: up	0.01	0.82	0.423
	Response direction: up	0.02	2.00	0.051
	Learn. cont.: up $\times$ resp. dir.: up	− 0.02	− 1.84	0.067

The “down” conditions serve as baseline conditions

#### Explicit judgment task

Participants’ responses by learning context are depicted in Fig. 1. A generalized linear mixed effect model was estimated for the proportion of “upwards” responses, containing only an intercept and random intercepts as well as random slopes for the learning context for both participants and items. A model that additionally contained a fixed effect for learning context predicted the participants’ answers significantly better than this baseline model ($\chi ^2(1) = 16.71$, $p < 0.001$, $\beta = -3.49$, $z = -6.33$). As can be seen in Fig. 1, both conditions significantly deviated from guessing probability in the expected direction.

### Discussion

In line with the results by Günther et al. (2018), we observed no action-congruency effect for words learned purely from language even though participants were clearly able to indicate the words’ implied locations when explicitly asked to do so. This result is surprising under the assumption that sleep between learning phase and test phase should lead to a consolidation of memory. However, participants in this study in principle had the possibility to largely ignore the learning material presented to them in the evening, and to only read it during the repetition phase in order to produce the results observed in the explicit judgment task. This leaves open the possibility that no consolidation during sleep has actually taken place. We addressed this issue in Experiment 2.

## Experiment 2

Experiment 2 was a modified version of Experiment 1: On the one hand, the learning phases now included control questions about the learned concepts which the participants had to answer correctly in order to complete the learning phase. On the other hand, we now included a second learning phase two days before the test phase, so that participants had more experience with the concepts and more opportunity to consolidate memory.

### Method

#### Participants

Data was collected from 45 native German speaking participants, 38 female and 7 male, all right-handed, $M_{{\text {Age}}} = 22.2$ years, SD$_{{\text {Age}}} = 3.58$ years. Data from one additional participant was excluded due to technical errors, and data from six additional participants due to high error rates.

#### Materials and procedures

The material, sleep phase, repetition phase, test phase, and explicit judgment task were identical to Experiment 1. However, in Experiment 2, we employed an extended learning phase. First, participants now performed the learning phase on the day before the lab session and also on the day before that, resulting in two identical learning phases. Second, we now included control questions in the learning phase. After reading all eight texts in random order (which were identical to Experiment 1), participants were presented with control questions—clozes such as *“The artificial sun which is fixed on a dome above a city is called *[ ]”, where they had to fill in the novel word labels learned before. The eight different questions were presented in random order. Participants received feedback for their answers. If not all of their answers were correct, participants were again presented with all learning texts, followed by all control questions. This was repeated until all answers were correct.^3^ We checked that participants did not quit the learning phases before testing them in the lab sessions.

### Results

#### Test phase

**Fig. 2:**
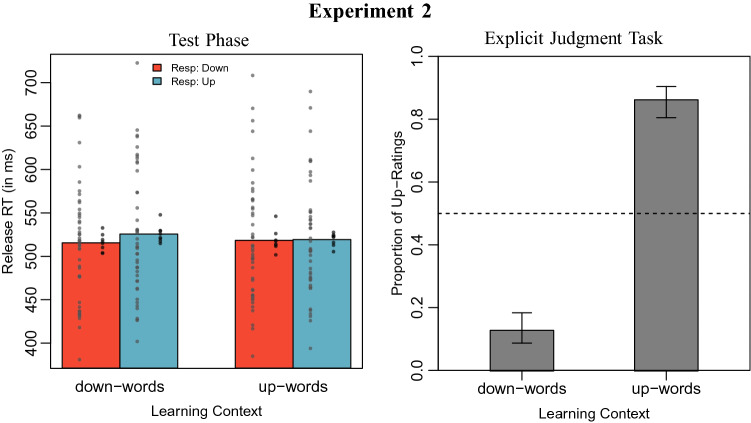
*Left panel* Mean release times in Experiment 2, by learning context and response direction. The grey circles on the left of each bar display mean reaction times by participants, the black circles on the right of each bar display mean reaction times by item. *Right panel* Proportion of upwards-location ratings for novel words by learning context, with 0.95 confidence intervals

Error trials (2.3 %) were excluded from the analysis. There were no trials with response times under 100 ms. Mean response times by learning context and response direction are displayed in Fig. 2.

We performed the same mixed-model analysis as described in Experiment 1. The model including an interaction between learning context and response direction did not perform significantly better in explaining the data than the model without it ($\chi ^2(1) = 3.34, p = 0.067$). We obtained a BIC-approximated Bayes factor of BF $= 0.0503$, indicating that the data are about 20 times more likely under the baseline model (positive evidence in favor of the baseline model). The model parameters for this model are reported in Table 1.

Since the *p* value of this analysis was quite close to 0.05, we ran an additional backup analysis before jumping to conclusions about the absence of a congruency effect. To this end, we additionally conducted an alternative one-factorial analysis in which the two experimental factors were merged into the single factor “congruency” (the up-up and down-down condition being the congruent conditions, the other two being the incongruent ones). In the mixed-model analysis (with the model including random intercepts and random slopes for congruency for both participants and items), including a fixed effect for congruency did not significantly improve the model ($\chi ^2(1) = 1.49, p = 0.222$). Again, for both types of analysis presented here, the same pattern emerged when restricting the analysis to items for which participants gave the correct answer in the explicit judgment task.

#### Explicit judgment task

Participants’ responses by learning context are depicted in Fig. 2. We employed the same baseline GLMEM as in the previous study, containing only an intercept and random intercepts as well as random slopes for the learning context for both participants and items, to predict the proportion of “upwards”-responses. A model that additionally contained a fixed effect for learning context predicted the participants’ answers significantly better than this baseline model ($\chi ^2(1) = 32.63$, $p < 0.001$, $\beta = -9.93$, $z = -3.97$). Again, both conditions significantly deviated from guessing probability in the expected direction (see Fig. 2).

### Discussion

In these first two experiments, we observed no automatic action-congruency effects for words learned purely from language even though participants were clearly able to indicate the words’ implied locations when explicitly asked to do so (in line with Günther et al., 2018). Notably, this was the case even though participants had far more experience with the novel words compared to these previous studies—we employed substantially extended learning phases, where the novel words described concepts central to sensible, coherent texts which participants read twice—and even though the association between the experiential traces could be consolidated in memory during sleep (Walker & Stickgold, 2006).

It can of course be argued that participants still had relatively little experience with these words, and therefore did not automatically access their meaning during reading. Participants also never encountered the novel words outside their learning contexts, which clearly described specific vertical locations. Therefore, they never had to use the words as retrieval cues for any sensorimotor information, resulting in weak associative links. In addition, participants never actively used these novel words in communication, and never encountered them outside an artificial lab setting. They might thus perceive them as obviously artificial experimental material with no real-world relevance, and not consider them as actual lexicon entries.

## Experiment 3

To address these issues, in Experiment 3 and 4, we moved from investigating novel words towards vertically associated familiar words whose referents participants never experienced directly (such as *Hades* or *pterosaur*). Thus, we employed words that are established lexicon entries and were not just recently learned in an artificial lab setting. We can therefore assume that participants have repeatedly encountered and used these words in natural communication settings, and to have a clear meaning representation of the described entities (including their vertical location).

### Methods

#### Participants

Following the results of the power analysis, we tested 45 participants (all right-handed; 36 female, 9 male; $M_{{\text {Age}}} = 23.5$ years, SD$_{{\text {Age}}} = 6.30$ years.). Data from one additional participant was excluded due to high error rates.

#### Materials and procedures

To create the item material, we collected rating data from 25 participants who did not participate in the actual study. For a set of 62 words, participants indicated on 5-point scales (a) the typical vertical locations associated to the word’s referents (from *very low* to *very high*), and (b) how much direct experience they made with the word’s referents during their lifetime (from *no experience* to *very much experience*). The 62 items were selected because we expected them to cover the entire range for all collected variables, including some filler items; the entire item list can be found at https://osf.io/vxrhn. Participants were explicitly instructed that depictions of the referents, for example in pictures and movies, can be counted as direct experience.

We selected eight words that were clearly associated with a vertical location, but with which participants indicated very little or no direct sensorimotor experience (see Table 2). Apart form the word material, the procedure of Experiment 3 was identical to the Test Phase of Experiment 1 and 2.

**Table 2:** Item material for Experiment 3 and 4, including mean ratings for the vertical location (1 = very low, 5 = very high) and amount of direct experience (1 = no experience, 5 = very much experience) for Experiment 3 and vertical location, amount of direct and indirect experience (1 = no experience, 5 = very much experience), and the percentage of participants who knew the word for Experiment 4

Word		Exp. 3		Exp. 4			
German	English	Location	Experience	Location	Direct exp.	Indirect exp.	% Known
Goldmine	Goldmine	1.36	1.60	1.57	1.14	2.67	100
Erdkern	Earth’s core	1.36	1.71				
Hades	Hades	1.84	1.72				
Plankton	Plankton	1.40	1.80				
Pottwal	Sperm whale			1.60	1.20	2.47	100
Tauchroboter	Diving robot			1.14	1.27	2.45	100
Fallgrube	Pitfall			1.80	1.27	2.60	100
Flugsaurier	Pterosaur	4.32	1.80	4.46	1.15	2.62	87
Supernova	Supernova	4.25	1.54				
Pegasus	Pegasus	4.04	1.80				
UFO	UFO	4.84	1.92				
Jupiter	Jupiter			4.60	1.47	2.67	100
Albatros	Albatross			4.05	1.62	2.43	100
Gigant	Giant			4.06	1.33	2.72	100

### Results

**Fig. 3:**
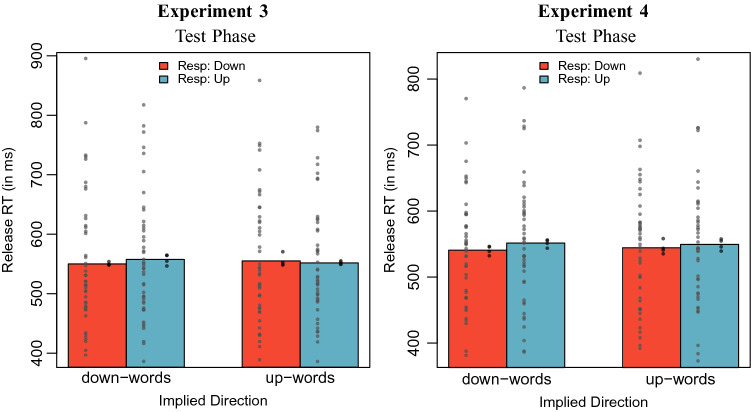
Mean release times in Experiment 3 (left panel) and Experiment 4 (right panel), by implied direction and response direction. The grey circles on the left of each bar display mean reaction times by participants, the black circles on the right of each bar display mean reaction times by item

The data were analyzed using the procedure described for the Test Phase analysis of Experiment 1. Error trials (3.3 %) and one overly fast trial were excluded from the analysis. Since we used real words, the factor *learning context* was replaced with *implied location*. Mean reaction times by implied location and response direction are displayed in Fig. 3 (left panel).

We performed the same model comparison as described in the previous analyses, except that the factor “learned direction” was now replaced by “implied direction”. The model including the fixed effect for the two-way interaction between response direction and implied location did not perform better than the model without this interaction, as indicated by a likelihood-ratio test ($\chi ^2(1) = 1.80, p = 0.180$). We obtained a BIC-approximated Bayes factor of BF $= 0.0233$, indicating that the data are about 43 times more likely under the baseline model (strong evidence in favor of the baseline model). The model parameters for the model including the interaction are reported in Table 3.

**Table 3:** Model parameters for the model including a fixed effect for the interaction between learning context and response direction in Experiment 3 and Experiment 4

Experiment	Parameter	$\beta$	*t*	*p*
Experiment 3	Intercept	6.26	225.47	< 0.001
	Implied location: up	0.01	1.06	0.327
	Response direction: up	0.01	1.33	0.198
	Impl. loc.: up $\times$ resp. dir.: up	− 0.02	− 1.42	0.194
Experiment 4	Intercept	6.26	270.27	< 0.001
	Implied location: up	0.00	0.34	0.745
	Response direction: up	0.02	1.23	0.235
	Impl. loc.: up $\times$ resp. dir.: up	− 0.01	− 0.38	0.718

### Discussion

Even though we employed real words as item material, we again observed no action-congruency effect. Thus, the factors discussed as potentially leading to the absence of this effect for novel words in Experiment 1 and 2—the limited learning experience with these novel words, the fact that participants never used them as a cue to retrieve sensorimotor experience, or that they never used or encountered them in natural contexts—do not offer a sufficient explanation for this absence.

Interestingly, even some limited experience with the word’s referents seems insufficient to elicit action-congruency effects, as participants in the rating did not equivocally indicate that they have *no* experience with the referents—the ratings differ slightly from the minimum value. This raises the possibility that the action-congruency effects observed by Öttl et al. (2017) after exposing participants to the word referents were partly due to the high salience and recency of the sensorimotor experience.

## Experiment 4

At this point, there is a fairly simple alternative explanation for the results in Experiment 3: Some of the words were not particularly frequent (for example *Hades* or *supernova*), and (some) participants could have simply not known the words. In this case, one could not reasonably expect any congruency effects.

We thus replicated Experiment 3, while ensuring that participants indeed knew the words presented to them. In this context, we also considerably extended the rating study and explicitly differentiated between direct and “indirect” experience (for example, in pictures and movies), in order to obtain a maximally adequate item set for Experiment 4.

### Method

#### Participants

In this experiment, we tested 44 native German speaking participants (one fewer than required due to technical problems, 41 right-handed; 35 female, 9 male; $M_{{\text {Age}}} = 23.6$ years, SD$_{{\text {Age}}} = 4.24$ years). Data from five additional participants was excluded due to high error rates (see the previous experiments)

#### Materials and procedures

The material for Experiment 4 was obtained in a web-based rating study, using the *jsPsych* software (de Leeuw, 2015). We assembled a list of 348 items, and instructed participants (who did not participate in the actual experiment) to indicate, on 5-point scales, the vertical location of the described object, the amount of direct sensorimotor experience with the described object, and the amount of “indirect” sensorimotor experience (for example in pictures or movies). They were further given the opportunity to indicate that they didn’t know a word. The item material was selected to cover the whole range of value combinations of vertical location and amount of experience, and rating results indicated that this manipulation was successful. The entire item list can be found at https://osf.io/vxrhn.

The questionnaire was administered to 203 participants. Each participant was presented with 30 randomly selected items, resulting in between 10 and 34 ratings per word. As the up-words (down-words) for our experimental material, we selected four words that, on average, (a) received very high/very low location ratings, (b) received very low direct experience ratings, (c) received low indirect experience ratings, and (d) were known to most participants. Thus, the words and their referents were very familiar to participants (prevalence—the number of speakers knowing a word—is strongly correlated familiarity and word frequency; Brysbaert et al., 2019), but participants had little to no sensorimotor experience with the word referents. The selected items are displayed in Table 2. Apart from the word material, the material and procedure of the Experiment 4 test phase was identical to that of Experiment 3.

After the experiment, participants were handed a questionnaire and instructed to indicate for each of the eight words in the item material whether they knew the word or not, and if they did, the vertical location associated to the described object (up vs. down).

### Results

The data were analyzed as described for Experiment 3. Again, error trials (3.3 %) and one overly fast trial were excluded from the analysis. Mean reaction times by implied location and response direction are displayed in Fig. 3 (right panel). Again, the model including a two-way interaction fixed effect between response direction and implied location did not outperform the model without this interaction ($\chi ^2(1) = 0.14, p = 0.711$). We obtained a BIC-approximated Bayes factor of BF $= 0.0103$, indicating that the data are about 97 times more likely under the baseline model (strong evidence in favor of the baseline model). The model parameters for the model including the interaction are reported in Table 3. The pattern of results stays unchanged if we exclude from the analysis trials including words for which participants gave no answers or incorrect location judgments, or indicated that they did not know the word, in the post-test phase questionnaire ($7.2 \%$ of the data).

### Discussion

The results of Experiment 3 were replicated in Experiment 4, demonstrating that (a) we again find no evidence for an action-congruency effect for items participants have no direct sensorimotor experience with, and (b) that the absence of this effect is not due to participants not knowing the words presented to them. Interestingly, the results in Experiment 4 also indicate that low to moderate levels of indirect sensorimotor experience with the described objects are not sufficient to elicit an action-congruency effect. This can potentially be attributed to the fact that “depicted” objects are usually not experienced in the same vertical location as their “real” counterparts (movies and pictures are usually encountered directly in front of the observer, or on a display they are holding in their hands).

These findings demonstrate that just controlling for the associated vertical location (see Goodhew & Kidd, 2016) when selecting the item material for studies on congruency effects is insufficient: In such studies, the absence of congruency effects could also simply result from a lack of direct experience, and not necessarily the absence of sensorimotor activation in their respective experimental paradigms.

## General discussion

In four experiments, we tested whether speakers automatically activate sensorimotor experience in word processing when they have no direct experience available with the word’s referents. To this end, we employed words associated with a vertical location, and employed an experimental paradigm in which previous studies observed automatic motor congruency effects during word processing (Lachmair et al., 2011; Öttl et al., 2017; Thornton et al., 2013). In Experiments 1 and 2, participants learned novel words and their associated vertical locations in several separate learning phases, with an intervening memory consolidation via night’s sleep. In Experiment 3 and 4, we employed familiar, vertically-associated words whose referents participants did not experience directly. In line with previous results by Günther et al. (2018), we did not observe automatic action-congruency effects for words learned from language alone in any of the experiments, even though participants in the present set of studies made far more experience with the words in richer linguistic learning contexts, and even though they had the opportunity for memory consolidation during sleep.

### Discussion of alternative explanations

This pattern of results is not a consequence of using only a limited number of eight items: In a pilot experiment, Günther et al. (2018) observed an action-congruency effect for a set of eight real words for which direct experience available (*cloud* or *basement*), the same number as items in the present experiments. It is also not a consequence of using novel words *per se*: Öttl et al. (2017) observed the effect for their set of eight novel words for which direct experience with the referents was available. Furthermore, Günther et al. (2020) also observed a congruency effect using a set of eight words, both for real words (Experiment 1) as well as novel words (Experiment 2). By following the results of an earlier power analysis (see Günther et al., 2018), it is also unlikely that the absence of an effect in four different experiments results from insufficient statistical power (even if the power estimate of 0.90 was an extreme over-estimation and the true power of each of our experiments was only at 0.50, the probability of not finding an existing effect would only be at $(1 - 0.50)^4 = 0.0625$ across all four studies). Finally, the absence of an effect can also not be ascribed to participants not understanding the words or not associating them with a vertical dimension, as indicated by the explicit judgment task in Experiment 1 and 2 and the rating results in Experiment 3 and 4.

In principle, there is also the possibility that we did not find a congruency effect as a result of the specific properties of the item material and the required response: In all experiments, participants had to react with upwards or downwards hand movements, while interactions with the referents of the presented words would not necessarily involve such vertical hand movements (take, for example, an underground city or artificial sun in Experiments 1 and 2, or *earth’s core * or *Jupiter* in Experiments 3 and 4). However, previous studies have shown that this word-level congruency effect is also consistently found for items such as *plateau*, *planet*, *sky*, *cloud*, or *skyscraper* on the one hand, and *swamp*, *submarine*, *basement*, or *underground* on the other hand (Lachmair et al., 2011)—all entities that we arguably don’t interact with more using vertical hand movements than the concepts in question. We thus don’t consider it likely that specifically this property of the item material used in the present studies caused the absence of the effect. However, it might still be the case that automatic congruency effects are more likely for non-experienced concepts that would inherently afford such vertical movements, which can be investigated in future studies.

In the context of this argument, it is also important to note that the congruency effect investigated here is not the classical action-sentence congruency effect (ACE; Glenberg & Kaschak, 2002) found for sentences describing certain actions and specific movements; instead, it is a pure word-level effect. This is especially relevant as the reliability of the classical ACE has recently been called into question (Papesh, 2015), especially since a large multi-lab collaboration has failed to replicate it (Morey et al., in press). However, this debate on the ACE did not yet consider word-level effects, which have been reliably observed across many different studies (Dudschig et al., 2012, 2014a, b; Dudschig & Kaup, 2017; Lachmair et al., 2011; Öttl et al., 2017; Thornton et al., 2013; Vogt et al., 2019; see also the pilot study in Günther et al., 2018). In the studies where this word-level effect was not observed, this can either be attributed to missing saliency of the vertical dimension in both the stimulus and response set (Dudschig & Kaup, 2017) or, as in the studies presented here, to the specific word material (novel word labels for non-experienced referents; compare Günther et al., 2018). Given the apparent robustness of this word-level effect,^4^ we don’t consider it likely that the null results of the present study are the result of a general non-replicability.^5^

### Theoretical implications

Our results are in line with accounts postulating that language processing does not always entail the automatic activation of sensorimotor experience (see Lebois et al., 2015). This is often explained in terms of task demands, in that we only engage in sensory and motor processing when required by the task (Günther et al., 2020; Ostarek & Huettig, 2019). However, we need to extend upon this explanation: As demonstrated by previous studies, such automatic action-congruency effects also emerge when direct experience with word referents is available, irrespective of whether they are well-known familiar words (Lachmair et al., 2011) or newly-learned novel words (Öttl et al., 2017). On the other hand, when that direct experience is missing, we observe no such effects, neither for novel (Experiment 1 and 2) nor for familiar words (Experiment 3 and 4). Thus, sensorimotor experience *can* be automatically activated even when not required by the task, but only if direct experience with the referent is available and sufficiently strong links to the linguistic stimulus are established.

Taken together, we can thus identify factors leading to activation of sensorimotor experience during language processing. Previous research has shown that concepts can be linked to sensorimotor experience (Lachmair et al., 2011), and that these connections can be established directly through experience with the referent (Öttl et al., 2017), but also indirectly via language (Günther et al., 2020). Whether this information is activated in a given context then depends on to what extent this “sensorimotor schema” is made salient. In cases where the connection to sensorimotor experience is strongly established, which can for example result from direct referent experience, this information is salient by default and will thus be easily activated even if not required by the task (as in the original Stroop task; Stroop, 1935). However, even then the task (i.e., the context in which language processing takes place) has to make this “sensorimotor schema” at least minimally salient: When reducing the saliency of the vertical dimension in the item set (by including words not related to a vertical dimension) as well as the response set (by including horizontal in addition to vertical answers), the action-congruency effect observed by Lachmair et al. (2011) disappears (Dudschig & Kaup, 2017). On the other hand, even in cases where the connection to sensorimotor experience is weaker—for example, when direct referent experience is missing—it can still be made salient depending on the task at hand and the level of processing: Günther et al. (2020) observe action-congruency words for novel words learned from language alone in a plausibility judgment task for sentences, which necessarily requires meaning access and a simulation of the sentence contents. However, as demonstrated in the present study, indirect connections between words and sensorimotor experience provided via language are not by themselves salient enough to be spontaneously activated.

In principle, it might of course still be the case that the availability of direct referent experience is not the deciding factor at play here. For example, one could assume that sensorimotor simulations can play the role of actual direct experience: If participants consistently had to simulate actions including the newly-learned words, such as *You scratch your mende* when they learned that a *mende* is a *bionic foot*, these simulations could be sufficient to establish strong connections between the words and sensorimotor information. In fact, judging the plausibility of such sentences was the test phase employed by Günther et al. (2020). If we imagine an experimental setup where such plausibility judgments form the learning phase instead of the test phase, one might possibly expect to observe the automatic congruency effects that were absent in the present study. We leave such investigations to future research. Nevertheless, from present study we can conclude that neither (a) a memory consolidation phase via sleep nor (b) a rich linguistic learning context result in any measureable automatic activation of the sensorimotor meaning aspect of words learned from purely linguistic experience.

## Footnotes

1 We employed this dependent variable in line with all previous studies employing the same test phase paradigm (Günther et al., 2018; Lachmair et al., 2011; Öttl et al., 2017). All analyses reported here give the same results when analysing the time until an outer button was pressed.

2 The maximal model additionally including random slopes for the interaction of the fixed effects (Barr et al., 2013) did not converge.

3 Of course, participants can write down the answers, but this again requires active engagement with the learning material.

4 Provided that there are no potential unpublished null-effect studies that we are not aware of.

5 Notably, the critical dimension is another difference between typical investigations of the classical ACE (horizontal, except for a few cases such as Borghi et al. (2004)) and our paradigma (vertical), but it isn’t obvious how this would influence the reliability of the effects in question.
